# Outcome Measures of Fatigue in Adults With Cancer Receiving Radiation Therapy: A Scoping Review

**DOI:** 10.1155/oti/7068284

**Published:** 2026-06-01

**Authors:** Courtney Apostol, Danielle Hitch

**Affiliations:** ^1^ Occupational Therapy, Sunshine Hospital, Western Health, St. Albans, Victoria, Australia, westernhealth.org.au; ^2^ Occupational Science & Therapy, Deakin University, Geelong, Victoria, Australia, deakin.edu.au

**Keywords:** cancer, cancer-related fatigue, fatigue, outcome measures, radiation therapy

## Abstract

Cancer‐related fatigue (CRF) is a debilitating symptom experienced by many people undergoing radiation therapy, significantly impacting daily functioning and quality of life. Occupational therapists play a vital role in managing CRF, yet the lack of standardised outcome measures tailored to occupational therapy practice hinders effective assessment and intervention. This scoping review is aimed at identifying and mapping validated fatigue outcome measures suitable for outpatient radiotherapy settings and discussing their relevance to occupational therapy. Following the Arksey and O’Malley framework, a comprehensive search was conducted across MEDLINE, CINAHL, Embase, and PubMed, identifying 445 records. After screening, 12 studies published between 2010 and 2022 from seven countries were included. Breast, head and neck and prostate cancers were commonly studied, most often using prospective cohort designs. Most studies used multidimensional, self‐reported outcome measures, administered at multiple times throughout radiotherapy and at follow‐up. While these measures assessed physical, emotional and cognitive fatigue, few addressed activity or participation in daily life. The findings highlight a gap in outcome measures that comprehensively evaluate activity and participation. This gap limits their utility in tailoring interventions or demonstrating the impact of occupational therapy. This review underscores the need for developing or adapting CRF outcome measures that better align with occupational therapy goals to enhance clinical decision‐making and research in outpatient radiotherapy settings.

## 1. Introduction

Cancer has a profound impact on individual’s physical, emotional and functional well‐being, with many experiencing a wide range of distressing symptoms as a result of both the disease and its treatment. Among these, fatigue is one of the most reported and debilitating symptoms. Cancer‐related fatigue (CRF), in particular, is a pervasive and distressing form of tiredness that is not proportional to recent activity and is not relieved by rest [1]. It affects many adults with cancer, especially those undergoing radiation therapy (RT), with prevalence rates as high as 84% reported in this group [2]. CRF is distinct from everyday fatigue, often being more intense, persistent and disruptive to daily life and participation.

The multidimensional nature of CRF encompasses profound energy depletion, mental exhaustion, muscle aches (often alongside pain), emotional distress and sleep difficulties [3]. It can vary across the cancer journey, meaning its impact on meaningful occupations may evolve over time. Therefore, it causes many people significant disruption to quality of life (QoL) and daily functioning, posing important occupational challenges for outpatients striving to maintain participation in meaningful activities of daily life. This creates a disconnect between what occupational therapy is aimed at addressing and what current fatigue outcome measures capture. This issue is particularly relevant in the context of RT, where fatigue is both highly prevalent and often cumulative over the course of treatment.

### 1.1. RT and CRF

RT uses high‐energy radiation like X‐rays, gamma rays or charged particles to target and destroy cancer cells [4]. It may be administered using external beam radiation (where a machine directs radiation at the tumour), or internal methods (where radioactive sources are placed near or inside the tumour) [4]. RT may be used as a stand‐alone treatment or in combination with surgery, chemotherapy or immunotherapy. Patients often experience acute and severe fatigue during RT which significantly impacts on daily functioning and QoL and often persists long after treatment has concluded [5].

RT treatment for cancer may have a significant impact on the patients’ ability to participate in daily life, particularly for older adults [6]. Occupational engagement tends to decrease during treatment, with nearly half of patients not returning to baseline 1 month after RT [7]. However, the occupational impact on social roles and activities varies due to the individual circumstances of patients, adjuvant chemotherapy regimens and cancer type [8].

### 1.2. Occupational Therapy for CRF

Given the significant and often prolonged impact of RT‐related fatigue on daily functioning, occupational therapy is uniquely positioned to address these challenges through the assessment of activity and participation to inform tailored interventions that enhance occupational performance [9]. In this context, activity refers to the execution of tasks or actions by a person, while participation refers to involvement in life situations, consistent with the International Classification of Functioning, Disability and Health (ICF) framework [10]. Examples include self‐care, domestic activities, employment, social participation and engagement in meaningful occupations.

In this review, participation relevant content within CRF outcome measures referred to items assessing role performance, engagement in daily activities, social participation or the impact of fatigue on occupational functioning. This reflects the definition of the ICF definition of this term, which specifies involvement in life situations [10]. Participation may be evaluated through self‐reported role engagement, perceived ability to complete daily activities, social participation or observed occupational performance. All of these constructs are central to occupational therapy practice, which focuses on enabling people to participate in meaningful daily life despite illness or disability.

Emerging evidence highlights the promise of several occupational therapy interventions, with exercise, physical activity programmes and psychosocial interventions associated with reduced CRF and improved functional outcomes [11–13]. The evidence for aerobic exercise is strong, while more moderate evidence exists for other occupational therapy interventions [11]. Group‐based occupational therapy has also been found to increase occupational performance and decrease fatigue [14].

Energy conservation and activity pacing interventions are also utilised as part of broader fatigue management approaches by occupational therapists to support people experiencing CRF. A pilot study of energy conservation training followed by telephone monitoring sessions [15] found significant improvement in only the sensory aspects of fatigue and evaluated its efficacy as modest. All of these interventions may be combined with educational programmes, relaxation techniques, problem‐solving strategies, environmental modifications and strategies for managing breathlessness as part of a package of rehabilitation or care [16, 17].

### 1.3. Outcome Measures for CRF

To effectively deliver and evaluate these interventions, occupational therapists require appropriate assessment methods and outcome measures for CRF. The National Comprehensive Cancer Network (NCCN) [1] United States definition of CRF encompasses physical, emotional, cognitive and spiritual dimensions. However, current measures inconsistently evaluate variables such as fatigue occurrence, severity or specific domains. Recent recommendations from Gentile et al. [18] noted that no currently available measures encompass all domains of the NCCN definition, and their psychometric qualities are also less than optimal.

Of relevance to occupational therapists is the ability of these outcome measures to evaluate activity and participation. The Fatigue Severity Scale (FSS) includes 2 out of 9 items about participation in activities of daily living [19], while 4 out of 20 items on the Multidimensional Fatigue Inventory (MFI) [20] assess reduced activity. The Patient Reported Outcomes Measurement Information System, Fatigue–Short Form (PROMIS F‐SF) [21] only includes participation in social activities. All these scales are patient‐rated outcome measures, and it remains unclear what other forms of evaluation occupational therapists undertake when enabling patients to manage CRF.

This lack of standardisation complicates clinical decision‐making and research, particularly in the context of RT where fatigue is a common concern. For occupational therapists, the scarcity of outcome measures focused on meaningful engagement in daily activities, roles and routines creates a critical gap, which hinders their ability to monitor the impact of their interventions on enhancing their patients’ ability to maintain participation despite CRF. Despite the availability of multiple measures, their limited focus on activity and participation raises important questions about their suitability for occupational therapy practice, particularly in time‐pressured clinical settings.

### 1.4. Rationale for This Review

The focus on RT in this study reflects both its prominence in clinical referrals for CRF and the structured, outpatient nature of treatment delivery, where fatigue is routinely monitored over time. In this context, occupational therapy input is often brief, sometimes limited to a single session, which places greater emphasis on efficient and targeted assessment. As a result, outcome measures need to be both practical and sufficiently sensitive to capture meaningful changes in activity and participation within a limited timeframe. RT also provides a relatively consistent clinical context in which fatigue follows a cumulative and predictable trajectory, supporting comparability across studies of outcome measure use.

The focus on RT was therefore driven by the need to translate the review findings directly into practice. CRF has a significant effect on adults undergoing RT, and occupational therapy plays a critical role in maintaining participation and QoL for these patients. The use of outcome measures which were not developed from an occupational perspective may be a barrier to gathering robust data that enables the monitoring of intervention effectiveness, informs the tailoring of interventions to individual needs and demonstrates the impact of occupational therapy interventions. In contrast, non‐RT treatment pathways such as surgery, chemotherapy or immunotherapy may involve substantially different fatigue trajectories, symptom patterns, treatment schedules and rehabilitation needs. Including these broader cancer populations would have introduced significant clinical heterogeneity, limiting comparability across studies and reducing the applicability of findings to outpatient RT practice.

A preliminary search of MEDLINE, the Cochrane Database of Systematic Reviews and JBI Evidence Synthesis was conducted to explore current evidence on this issue but found no existing or ongoing systematic or scoping reviews. This scoping review is aimed at systematically identifying and mapping validated fatigue outcome measures suitable for outpatient RT settings, with a particular emphasis on their relevance to occupational therapy practice. By synthesising the available evidence, the review sought to bridge the gap between current assessment tools and the practice needs of occupational therapists, ultimately enhancing their capacity to assess and manage CRF effectively.

The review question guiding this investigation is as follows: What validated outcome measures are available to guide occupational therapists working with people receiving RT and experiencing CRF?

## 2. Methods

This scoping review proceeded in accordance with the methodological framework outlined by Arksey and O’Malley [22], as adapted by the Joanna Briggs Institute [23]. This approach involved five sequential steps: (1) identifying the research question, (2) identifying relevant studies, (3) selecting studies, (4) charting the data and (5) collating, summarising and reporting the results. A scoping review methodology was chosen due to its ability to map expansive and multifaceted evidence bases and pinpoint evidence gaps for future development. Ethics approval is not required, as this review includes previously published literature. The protocol for this review was registered on the Open Science Framework prior to its commencement (10.17605/OSF.IO/9XBQK) [24].

### 2.1. Eligibility Criteria

The eligibility criteria address the population, intervention, outcome and publication characteristics (see Table 1).

**Table 1:** Review eligibility criteria.

Criteria	Rationale
Adults (≥ 18 years) with confirmed cancer diagnosis undergoing RT or combined chemoradiation therapy	Focus on the adult population receiving relevant treatment to ensure applicability of findings to this group
Outcome measures previously validated to assess cancer‐related fatigue (CRF) and applied in cancer populations	Ensures use of established tools for CRF assessment. Measures not originally developed for cancer populations were included where they had been applied in studies involving people with cancer to reflect real‐world clinical and research use
Outcome measures with a described structure, domains or item content related to fatigue	Ensures sufficient detail is available to examine what aspects of fatigue are assessed, including relevance to activity and participation
Outcome measures applied in a clinical practice setting	Focuses on practical utility of outcome measures in real‐world outpatient RT contexts
Published in English	Accommodated the language abilities of the review team
Primary studies published after 1 January 2010	Ensures currency and relevance of findings to contemporary clinical practice
Evidence from any country and outpatient RT context	Anticipated broad relevance to most patients receiving RT globally
Exclusion of studies where fatigue is not the primary focus (e.g., studies focused on pain, QoL, psychological distress or psychosocial factors), or where fatigue is examined outside the context of cancer	Ensures focus remains specifically on CRF during RT, avoiding confounding symptoms or unrelated fatigue conditions
Exclusion of studies exploring fatigue as a secondary outcome related to other variables	Maintains focus on outcome measures designed and validated primarily for CRF assessment to ensure direct relevance and translatability to clinical practice

The focus on RT and chemoradiotherapy contexts was intended to ensure comparability across outpatient settings where fatigue is routinely monitored. Other multimodal treatment pathways were not specifically included due to greater variability in fatigue presentation and timing; therefore, this restriction was intended to reduce heterogeneity in fatigue experiences and support more meaningful comparison of outcome measure use across studies.

### 2.2. Search Strategy

A comprehensive search strategy was completed to identify both published and unpublished studies (such as preprints). A draft search strategy was developed in consultation with a specialist hospital librarian and a limited search (see Supporting Information) conducted in MEDLINE to refine keywords and Medical Subject Headings (MeSH) terms. These terms were then adapted for the final search conducted on 26 March 2025, including studies published up to 31 December 2024 across four databases: MEDLINE, CINAHL, Embase and PubMed. The search used terms such as ‘cancer’, ‘fatigue’, ‘cancer‐related fatigue’, ‘radiation therapy’ and ‘outcome measures’, combined with Boolean operators. The reference lists of included studies were also searched using the Connected Papers platform [25], with three further potentially eligible studies identified.

Grey literature sources, such as reports, theses and conference proceedings, were not included because the review focused on peer‐reviewed primary studies reporting validated CRF outcome measures applied to clinical RT settings. This decision was made to maintain a focused and replicable search strategy, although it may have excluded emerging tools or unpublished clinical evaluations.

### 2.3. Study Selection

Citations identified through the search were imported into the Covidence platform [26], where duplicates were removed using both automated and manual processes. Two reviewers (C.A. and D.H.) screened titles and abstracts against the eligibility criteria. Potentially relevant studies were retrieved in full text and reassessed against the criteria by the same reviewers, with a small number of discrepancies (*n* = 12, 3.9%) resolved through discussion and consensus. All reasons for exclusion at the full‐text stage were documented, and the overall selection process was reported using a Preferred Reporting Items for Systematic Reviews and Meta‐Analyses extension for Scoping Reviews (PRISMA‐ScR) flow diagram [27].

### 2.4. Data Extraction

Data was extracted by two independent reviewers (C.A. and D.H.) using a customised extraction tool developed within Covidence [26]. Extracted data included study characteristics (e.g., author, year and design), participant details (e.g., sample size and cancer type), context (e.g., outpatient RT setting) and outcome measure characteristics (e.g., name, domains, item content and administration method). Particular attention was given to whether outcome measures included items related to activity, participation or role performance to assess their relevance to occupational therapy practice. This included examining the extent to which outcome measures captured aspects of activity and participation in daily life, as well as their feasibility and suitability for use in brief outpatient RT contexts. The potential usefulness of outcome measures for real‐world outpatient RT practice was informed by factors such as administration method, burden and relevance to activity and participation.

### 2.5. Data Analysis and Presentation

Consistent with scoping review methodology, formal quality appraisal was not undertaken because the purpose of this review was to map the range, characteristics and occupational therapy relevance of available outcome measures, rather than to evaluate intervention effectiveness or synthesise findings according to methodological quality. However, the lack of formal assessment of research rigour should be considered when interpreting the findings.

Data was analysed descriptively, using numerical summaries to detail the quantity, design and characteristics of included studies. A descriptive thematic analysis was also undertaken to identify key themes and concepts related to CRF outcome measures. All results are presented in Tables 2 and 3 accompanied by a narrative synthesis to highlight patterns, gaps and implications for occupational therapy practice and future research.

**Table 2:** Characteristics of included studies.

Study citation and country	Aim/s	Design	Recruitment/sample	Inclusion/exclusion	Type of cancer
Abel et al. [28] Sweden	Analyse patient‐reported fatigue in patients with HNC receiving RT and explore possible association with organ‐at‐risk doses	Prospective cohort study	Discussed at a regional tumour board *n* = 126 29 F (23%); mean age: 60	Inclusion: patients with newly diagnosed HNC referred for curative RT from 2008 to 2010. Exclusion: not documented	HNC Oral *n* = 16 Oropharynx *n* = 80 Hypopharynx *n* = 9 Nasopharynx *n* = 11 Unknown primary *n* = 10
Andic et al. [3] United States	Determine RT‐related changes in CRF across 3 timepoints and identify optimal MFI‐20 thresholds warranting intervention	Prospective cohort study	Clinic patients *n* = 88 88 F (100%); age range: 26–75	Inclusion: surgery with or without neoadjuvant/adjuvant chemotherapy followed by RT with or without hormone therapy. Exclusion: history of major psychiatric disorders or substance abuse/dependence in past 12 months	Breast
Cheon et al. [29] Canada	Examine changes in fatigue scores for patients receiving RT for bone metastases and its impact on QoL	Prospective cohort study	Rapid response RT program patients *N* = 881; Group 1: ESAS *n* = 399; 191 F (48%); mean age: 68; Group 2: EORTCQLQ‐C30 or EORTCQLQ‐C15‐PAL n = 482 331 F (69%) Mean age: 60	Inclusion: documented fatigue scores at baseline and 1 month follow‐up. Exclusion: not documented	Bone metastases
De Paula et al. [30] Brazil	Identify fatigue frequency and affected domains in patients with HNC undergoing RT, at the beginning, middle and end of treatment	Longitudinal and prospective study of quasiexperimental design	Clinic patients *n* = 60; 7 F (12%); age groups: 18–20 (*n* = 2, 3%), 21–40 (*n* = 27, 45%) 41–60 (*n* = 28, 47%), 61–80 (*n* = 3, 5%)	Inclusion: 18+ years, HNC diagnosis, at beginning of RT. Exclusion: unable to answer simple questions	HNC
Dickinson et al. [31] United States	Describe the multidimensional fatigue experience of men with prostate cancer before, during and after RT	Descriptive longitudinal study	Clinic patients *n* = 57 (*n* = 47 RT + ADT), n = 10 = RT only; 57 M (100%); mean age: 66	Inclusion: 21+ years, scheduled to receive ERBT exclusion: inflammatory condition, infection, other types of cancer, history of major psychiatric disorders or substance abuse/dependence in past 5 years, chemotherapy before EBRT, taking steroids, nonsteroidal anti‐inflammatories or tranquilizers	Prostate
Fransson [32] Sweden	Determine the frequency, severity and changes in fatigue during external beam RT and up to 5 years afterward and whether this was a predictor for increased fatigue during treatment	Prospective cohort study	Clinic patients *n* = 407 407 M (100%); mean age: 66	Inclusion: prostate cancer diagnosis. Exclusion: not documented	Prostate
Hauth et al. [33] Germany	Investigate the impact of RT on CRF and overall QoL in breast cancer patients	Prospective cohort study	Clinic patients *n* = 66 66 F (100%); median age: 57	Inclusion: breast cancer diagnosis, indication for adjuvant curative RT, completion of baseline and follow‐up outcome measures. Exclusion: not documented	Breast
Lam et al. [2] Canada	Identify trends and risk factors in patient‐reported fatigue associated with breast RT	Prospective cohort study	Clinic patients *n* = 651 651 F (100%); median age: 59	Inclusion: ESAS completed before or during RT. Exclusion: not documented	Breast
Poirier [34] United States	Identify the impact of fatigue, site‐specific side effects and individual characteristics on role activities during RT	Secondary data analysis	Clinic patients *n* = 77 45 F (58%); mean age: 54	Inclusion: minimum 4 weeks of curative or adjuvant RT, Karnofsky Performance Status Scale [KPSS] > 70, working at the time of cancer diagnosis. Exclusion: receiving brain RT or for palliative intent, coexisting unstable medical or psychiatric diagnoses	Multiple breast *n* = 34; lung *n* = 13 HNC *n* = 10; abdomen/pelvis *n* = 9
Pulenzas et al. [35] Canada	Determine changes in fatigue score following whole brain RT	Retrospective cohort study	Clinic patients *n* = 264; Group 1: ESAS and BASIQ. *n* = 36 22 F (61%); mean age: 64; Group 2: SQoLI, EORTC QLQ‐C15‐PAL, BN20+2, EORTCQLQ‐C30 and FACT‐G. *n* = 228 77 F (34%); mean age: 63	Inclusion: only receiving WBRT. Exclusion: patients receiving other treatments	Metastatic brain
Raju et al. [36] India	Assess fatigue in cancer patients who receive RT	Quantitative descriptive research design	Admitted and community RT patients. *n* = 138 70 F (51%); age range: 20–65	Inclusion: aged 20–65 years, any malignancy diagnosis, receiving RT only. Exclusion: not documented	Multiple reproductive *n* = 50; gastrointestinal *n* = 20; respiratory *n* = 4; haematological *n* = 3; HNC *n* = 61
Reidunsdattter et al. [37] Norway	Explore whether modern RT, alone and in combination with adjuvant treatments, influenced the level and the course of fatigue in breast cancer patients during RT and over 12 months	Prospective longitudinal study	Clinic patients *n* = 245 (*n* = 231 at follow‐up) 245 F (98%); mean age: 58	Inclusion: referred for postoperative local or locoregional RT either alone or in addition to CT, no metastatic disease, no physical or psychological disorders that would interfere with participation, able to speak and understand Norwegian. Exclusion: patients who developed metastatic disease during follow‐up	Breast

Abbreviations: ADT = androgen deprivation therapy, BASIQ = Brain Symptom and Impact Questionnaire, BN20 +2 = European Organization for Research and Treatment of Cancer Quality of Life Questionnaire—Brain Neoplasm, CRF = cancer‐related fatigue, CT = chemotherapy, EORTC QLQ‐C15‐PAL = The European Organisation for Research and Treatment of Cancer Quality of Life Questionnaire—Core 15 Palliative, EORTCQLQ‐C30 = The European Organisation for Research and Treatment of Cancer Quality of Life Questionnaire—Core 30, ESAS = Edmonton Symptom Assessment System, F = female, FACT‐G = Functional Assessment of Cancer Therapy—General, HNC = head and neck cancer, M = male, MFI = Multidimensional Fatigue Inventory, QoL = quality of life, RT = radiotherapy/radiation therapy, SQoLI = Spitzer Quality of Life Index, U/K = unknown.

**Table 3:** Outcome measures.

Study citation and country	CRF tool	Dim.	Original population	# fatigue items/domains	Administration in study	Other tools
Abel et al. [28] Sweden	EORTC FA12	Multi	Diverse cancer patients	12 items, 3 domains—physical, emotional and cognitive fatigue	Mailed for completion at baseline and 1, 3, 6, 12, 24 and 60 months after RT	EORTC QLQ‐C30
Andic et al. [3] United States	FIR	Uni	*FIR* not reported	*FIR* 1 item (1–3 mild, 4–6 moderate, 7–10 severe)	Method of administration unclear. Both outcome measures completed at baseline, last week of RT and 6 weeks post RT	N/A
	MFI‐20	Multi	*MFI-20* breast cancer	*MFI-20* 20 items, 5 domains—general fatigue, physical fatigue, mental fatigue, reduced activity and reduced motivation		
Cheon et al. [29] Canada	ESAS	Multi	*ESAS* diverse cancer patients in palliative care	*ESAS* 2/9 items *EORTC QLQ-C15-PAL* 2/15 items	Completed in the clinic at baseline, 1, 2 and 3 months following RT	QLQ‐C30 ESAS—all other questions QCQ‐C15‐PAL—all other questions
	EORTC QLQ C‐15‐PAL	Multi	*EORTC QLQ-C15-PAL* diverse cancer patients in palliative care			
De Paula et al. [30] Brazil	RPFS	Multi	Breast cancer	22 items, 4 domains—behavioural/severity, sensory, cognitive/mood, affective	Administered by interview, at baseline, middle and end of RT	N/A
Dickinson et al. [31] United States	RPFS	Multi	Breast cancer	22 items, 4 domains—behavioural/severity, sensory, cognitive/mood, affective	Method of administration: medical records and self‐reported questionnaires. Administered at baseline, before RT initiation, RT midpoint, RT completion and at 1, 3, 6, and 12 months post‐RT	Hamilton Depression Rating Scale (HAM‐D) Patient‐Reported Outcomes Measurement Information System v1.0‐Sleep Disturbance (PROMIS‐SD)
Fransson [32] Sweden	EORTC QLQ‐C30	Multi	Diverse cancer patients	3/30 items	Administered in clinic initially and then by mail at later timepoints. Completed before RT, 3 months, 1 year, 3 years and 5 years post‐RT.	EORTC QLQ‐C30—all other questions
Hauth et al. [33] Germany	FACIT–fatigue	Multi	Diverse cancer patients	13/40 items (fatigue subscale only)	Administered in clinic before postoperative RT, end of RT and 10‐month follow‐up	FACIT‐G—all other questions
Lam et al. [2] Canada	ESAS	Multi	Diverse advanced cancer patients	2/9 items	Administered weekly in clinic visits or by follow‐up phone calls at baseline, weekly during RT, 6 weeks, 1 month and 3 months post‐RT	ESAS—all other questions
Poirier [34] United States	RPFS	Multi	*RPFS* breast cancer	*RPFS* 4/22 items	Administered in clinic at baseline, weekly during RT and 1 month post‐RT	N/A
	BFI	Uni	*BFI* diverse cancer patients	*BFI* 9 items		
Pulenzas et al. [35] Canada	ESAS	Multi	*ESAS* diverse cancer patients in palliative care	*ESAS* 2/9 items	Method of administration unclear as reused from previously conducted studies. Administered at baseline and follow‐up between 1 and 3 months post‐RT	All other questions on: ESAS BASIQ EORTC QLQ‐C30 EORTC QLQ‐C15‐PAL FACT‐G Spitzer Quality of Life Index (SQLI) EORTC Quality of Life‐Brain module (EORTC QLQ‐BN20+2)
	BASIQ	Multi	*BASIQ* brain metastases population	*BASIQ* 2/18 items		
	EORTC QLQ‐C30	Multi	*EORTC QLQ-C30* diverse cancer patients	*QLQ-C30* 3/30 items		
	EORTC QLQ‐C15‐PAL	Multi	*EORTC QLQ-C15 PAL* diverse cancer patients in palliative care	*EORTC QLQ-C15-PAL* 2/15 items		
	FACT‐G	Multi	*FACT-G* diverse cancer patients	*FACT-G* 2/27items		
Raju et al. [36] India	FAS	Uni	Fatigue in individuals with systemic lupus erythematosus (SLE) and general chronic conditions	10 items	Administered in the clinic, but timepoints were unclear	N/A
Reidunsdattter et al. [37] Norway	EORTC QLQ‐C30	Multi	Diverse cancer patients	*QLQ-C30* 3/30 items	Administered in clinic at baseline, end of RT, 3, 6 and 13 months post‐RT	N/A

Abbreviations: BASIQ = Brain Symptom and Impact Questionnaire (37), BFI = Brief Fatigue Inventory (38), Dim. = dimensionality, EORTC QLQ C‐15‐PAL = EORTC Core 15 Palliative (41), EORTC = European Organisation for Research and Treatment of Cancer (39), EORTC‐FA12 = EORTC Cancer‐Related Fatigue (40), EORTC‐QLQ‐C30 = EORTC Quality of Life Questionnaire 30 item (42), ESAS = Edmonton Symptom Assessment System (43), FACIT‐F = Functional Assessment of Chronic Illness Therapy–Fatigue (44), FACT‐G = Functional Assessment of Cancer Therapy‐General (45), FAS = Fatigue Assessment Scale, FIR = Fatigue‐Intensity Rating (46), MFI‐20 = Multidimensional Fatigue Inventory 20 (20), N/A = not applicable, RFS = Revised Piper Fatigue Scale (47).

## 3. Results

**Figure 1:**
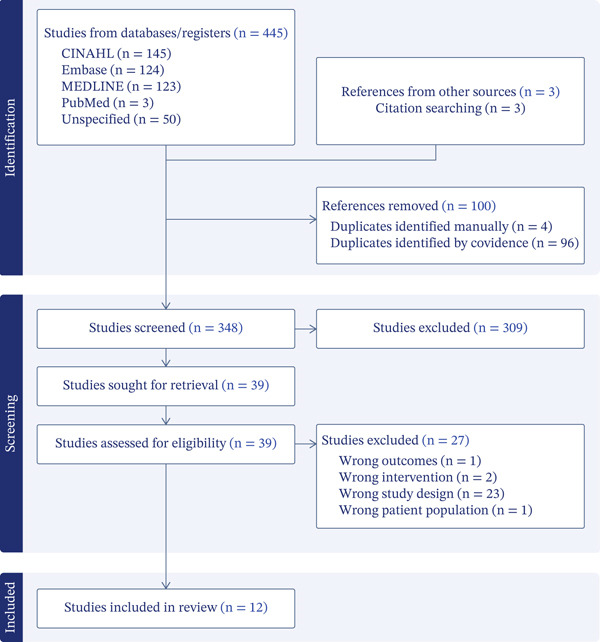
Study selection process [28].

The search and study selection process for this scoping review is summarised in the PRISMA‐ScR flow diagram (Figure 1). The search initially identified 445 records, from which 100 duplicates were removed (through a combination of automated and manual processes). Of the remaining studies, 39 were retrieved for full‐text review, with 12 ultimately meeting the inclusion criteria and being included in the final analysis. The primary reasons for exclusion at the full‐text stage included ineligible participant populations, outcome measures not validated for CRF or studies lacking application in clinical practice settings. Additional hand‐searching of reference lists via Connected Papers [25] identified three potentially eligible studies; however, all were screened out in the review process.

### 3.1. Study Characteristics

As displayed in Table 2, the included studies were published between 2010 and 2022 and originated from seven countries (United States and Canada *n* = 3; Sweden *n* = 2; Germany, Norway, Brazil and India *n* = 1). No eligible studies published after 2022 met the inclusion criteria. Half the published studies adopted a prospective cohort method (*n* = 6, 50%), and a further four (33%) utilised descriptive or longitudinal designs.

Sample sizes ranged between 36 and 881 participants, with a median of 88. Gender distribution was reported in 8 studies, showing a slight male predominance (47% female and 53% male across 1676 participants with gender data). Mean or median ages, where reported, ranged from 57 to 64 years. Inclusion criteria varied between studies, exclusion criteria were not always reported and the overall level of detail was inconsistent. All included studies recruited participants from clinical settings, the majority of which were drawn from general clinic populations. Breast cancer was the most frequently studied type (*n* = 5, 42%), followed by head and neck cancers (*n* = 3, 25%) and prostate cancer (*n* = 2*%*).

The aims of included studies most frequently assessed the nature and course of CRF (*n* = 9, 75%), with some also investigating factors which cause CRF (*n* = 5, 42%). Some looked at the links between CRF and function or QoL (*n* = 4, 33%), but only one [3] is aimed at informing clinical practice, and none specifically addressed occupational therapy practice.

### 3.2. Outcome Measures

Only six (46%) of the included fatigue measures or subscales included items related to activity or participation, operationalised as role performance, engagement in daily activities, social participation or occupational functioning, indicating limited alignment with occupational therapy priorities. Table 3 presents a summary of the outcome measures identified in the included studies which specifically targeted fatigue, along with those measuring other outcomes. Thirteen different fatigues tools were employed, including various formats, content and administration schedules. The majority were multidimensional outcome measures (*n* = 10, 83%) where fatigue was measured on a subscale or subset of questions.

Three tools (e.g., the Brief Fatigue Inventory [BFI], the Fatigue‐Intensity Rating [FIR] and the Fatigue Assessment Scale [FAS]) were unidimensional and measured fatigue as a single construct. Four multidimensional fatigue tools were identified: the European Organisation for Research and Treatment of Cancer Fatigue scale (EORTC FA12), the Multidimensional Fatigue Inventory 20 (MFI‐20), the Piper Fatigue Scale (PFS) and the Revised Piper Fatigue Scale (RPFS). These outcome measures offered insight into CRF across several domains, including general fatigue, physical fatigue, activity participation and cognition.

The populations in which these outcome measures were originally validated also varied. While some measures were developed for multiple cancer populations (such as the European Organisation for Research and Treatment of Cancer Quality of Life Questionnaire 30 item [EORTC QLQ‐C30]), others were originally designed for specific cancer or other populations. For example, the RPFS was originally validated with breast cancer patients, and the FAS was developed for people with systemic lupus erythematosus and other conditions but has also been applied in oncology settings.

Most measures were self‐reported and completed in clinical settings at multiple timepoints, usually baseline, during treatment and at follow‐up following RT. Some studies completed postal surveys [28, 32], while others utilised interviews to administer their tools [30]. Several included studies did not report how their outcome measures were administered. Most were administered alongside other outcome measures, which typically assessed overall QoL or symptom burden. Commonly coadministered tools were other tools included in the EORTC suite of outcome measures and the Edmonton Symptom Assessment System (ESAS). While administration methods varied, this did not substantially influence the extent to which outcome measures captured activity or participation.

The number of fatigue‐related items included on each outcome measure ranged from as few as 1 (FIR) to as many as 22 (RPFS), indicating significant diversity in how CRF is contextualised across the outcome measures.

Overall, multidimensional outcome measures appeared better suited to capturing the complexity of CRF across physical, emotional and cognitive domains, particularly in outpatient RT contexts where fatigue fluctuates over time. However, many of these tools were also longer and potentially less feasible for brief clinical encounters. In contrast, shorter unidimensional measures were more feasible for routine screening but provided limited insight into the functional or occupational impact of fatigue. Across both tool types, relatively few measures included meaningful assessment of activity, participation or role engagement, limiting their usefulness for occupational therapy assessment and intervention planning.

## 4. Discussion

A key finding of this review is that relatively few outcome measures capture activity, participation or role performance, despite these concepts being central to occupational therapy practice. This scoping review identified 12 studies that employed validated outcome measures to assess CRF in adults receiving RT, with a total of 13 unique tools utilised across diverse cancer populations and outpatient settings. Most of these measures were multidimensional, however item content varied, reflecting inconsistent CRF conceptualisation. Administration methods also differed, with no two taking the same approach. Differences in administration method (e.g., self‐report, interview and postal survey) appeared to determine perceived feasibility rather than the capacity of tools to capture participation or occupational engagement.

The findings of this review suggest that many existing CRF outcome measures adequately measure fatigue severity and multidimensional symptom burden, particularly across physical and emotional domains. However, an important conceptual gap remains between what these tools were designed to measure and the broader occupational impact of fatigue experienced by people undergoing RT. While several measures demonstrated strong symptom‐focused assessment properties, relatively few evaluated how fatigue affects participation in daily activities, social roles or meaningful occupations, despite these being central concerns for occupational therapy practice.

It is important to distinguish between the conceptual scope of outcome measures (i.e., the domains they are designed to assess) and their psychometric robustness (i.e., reliability, validity and sensitivity to change). Multidimensional tools align with the NCCN definition of CRF, which includes physical, emotional, cognitive and spiritual dimensions [1]. However, participation is rarely addressed (e.g., only 2/9 items in ESAS or 3/30 in EORTC QLQ‐C30) omitting an important variable and leaving them less comprehensive. Participation in meaningful occupations makes a fundamental contribution to overall QoL and is a key focus of occupational therapy practice. However, health professionals often underestimate its prevalence and importance to health and wellbeing [38]. Occupational therapists should consider how directly their chosen measure evaluates the links between activity, participation and CRF, and multidimensional fatigue‐specific outcome measures are recommended for holistic assessment.

For occupational therapy, the inclusion of participation reflects its central role in the profession’s clinical decision‐making. Understanding how fatigue affects engagement in daily activities, roles and routines enables occupational therapists to identify unmet needs, tailor interventions such as energy conservation and activity pacing and monitor meaningful outcomes over time. In practice, this focus can support more appropriate referrals to occupational therapy services, particularly for people facing significant challenges to participation despite a relatively stable clinical presentation. It may also inform interdisciplinary care planning by highlighting functional impacts that are not captured by symptom focused measures, encouraging a team‐based approach to support and rehabilitation.

In several included studies, fatigue was assessed with selected items from larger outcome measures designed to evaluate constructs such as QoL. This often reflects the pragmatic use of available tools within clinical practice and research, especially when fatigue is one of many outcomes of interest. However, selecting individual fatigue‐related items from these measures is problematic. While item‐level analysis can improve predictive performance in some cases [39], it may also reduce validity, reliability and sensitivity when items are removed from their original scale context [40]. It may also limit interpretability if these items may overlap with other constructs (e.g., pain or distress). If isolated items are used, occupational therapists should acknowledge they are proxy measures of fatigue and justify why validated fatigue‐specific tools were not feasible.

The most prevalent methodologies also indicate that these outcome measures are being used to track changes in CRF over time, which aligns with their use in clinical contexts [12]. However, the lack of experimental designs suggests that they are not currently applied to studies of intervention efficacy. Another methodological issue is the use of outcome measures which have not been validated on the cancer population to which they are applied, which may decrease their sensitivity to detecting fatigue in patients with other cancers. Stronger methods and validation would improve clinical utility.

In occupational therapy, CRF scales are regularly used for screening, and there is little available research into their use as outcome measures [41]. Given the prevalence of CRF, routine screening is recommended to avoid undertreatment and inform occupational therapy care plans [42]. CRF often accumulates over the course of RT particularly for older patients [43, 44], as illustrated by the longitudinal, repeated outcome measurement in the included studies. Tracking CRF over time provides valuable information for tailoring interventions like energy conservation and activity pacing to specific phases of treatment. Further research is needed to test these measures’ ability to detect intervention effects.

The review findings are broadly consistent with existing clinical guidance emphasising the importance of assessing fatigue in people undergoing RT [1, 42]. However, the variability in outcome measures identified in this review highlights ongoing challenges in selecting tools that are both feasible and aligned with occupational therapy priorities. Many tools have limited conceptual coverage of activity and participation, rather than this being primarily a limitation of their psychometric properties, which are generally robust. While several multidimensional measures demonstrate acceptable psychometric properties, their limited inclusion of activity and participation domains restricts their usefulness for occupational therapy practice. Measures requiring repeated administration or containing large numbers of items could provide richer multidimensional data but may be less practical in outpatient RT environments where occupational therapy contact is brief and fatigue burden is already high.

Observational evaluations, patient‐rated outcome measures including those evaluating sleep and rest‐activity cycles have been found to correlate with CRF severity [45]. However, the broad scope of items in the reviewed outcome measures (which often refer only to ‘daily life’) does not account for nuanced differences in the impact of fatigue on different occupations. They also limit opportunities to reflect on the full range of meaningful occupations and may therefore lead to an underestimation of the impact of fatigue. Occupational therapists should therefore use standardised tools alongside observation and performance measures to obtain a comprehensive understanding of the impact of CRF on occupational engagement.

Inconsistent reporting in the included studies limits interpretability of the evidence base. Gender was sometimes missing, and age is described using means, medians, age groups and ranges, both of which limit comparability between groups. Administration procedures are generally poorly described; for example, the content and format of the FIR [3] is unclear and not supported by other references. Occupational therapists must critically appraise measure validity and study quality when critiquing evidence for potential transfer to their practice.

### 4.1. Implications of the Findings

The findings of this scoping review highlight several practical considerations for occupational therapists using CRF outcome measures in their practice. Self‐reported tools appear to be feasible in outpatient RT contexts, but their coadministration with other measures may increase patient burden, particularly for people already experiencing fatigue. Unidimensional tools offer simplicity but lack the depth of exploration needed to inform tailored interventions. Multidimensional tools are more time‐intensive but are generally more suited to the requirements of high‐quality assessment. Cancer diagnosis can lead to significant distress and overwhelm, and patients early in their cancer journey may struggle to fully participate in their care [46]. Occupational therapists must balance the advantages and disadvantages of each approach to find tools which enable a focus on activity and participation while also remaining practical for clinical use.

From an occupational therapy perspective, fatigue outcome measure selection should be guided not only by symptom measurement properties but also by the extent to which tools capture the impact of fatigue on daily activities, occupational roles and participation. In brief outpatient RT settings, shorter multidimensional measures may provide a practical balance between feasibility and clinical usefulness, particularly where they include items relating to activity or participation. However, occupational therapists may also need to supplement fatigue‐specific tools with broader occupational performance or participation measures to fully understand the functional impact of CRF and guide intervention planning. Identifying participation restrictions associated with fatigue may help occupational therapists prioritise interventions such as energy conservation, activity pacing, environmental modification, role adaptation and support for maintaining engagement in meaningful occupations. Outcome measures incorporating activity and participation domains may also assist occupational therapists to monitor functional change over time and evaluate the impact of interventions beyond symptom severity alone.

The absence of a gold standard for CRF assessment, as noted by Gentile et al. [18], remains a challenge. While tools like the MFI‐20 and FSS include some items related to activity and participation, no measure fully aligns with the multidimensional definition of CRF proposed by the NCCN or comprehensively addresses occupational therapy’s focus on functional outcomes. This underscores the need for future development of CRF outcome measures that prioritise functional outcomes, such as engagement in daily activities and social roles, to better support clinical decision‐making and intervention planning in RT settings. The triangulation of multiple forms of assessment (i.e., patient rated, observational and objective) at various stages of RT treatment and follow‐up remains the recommended approach for now.

### 4.2. Limitations

Limitations related to both the included studies and the scoping methodology itself should be considered when interpreting these findings for practice. Geographical and cancer type biases in the studies highlight significant gaps in the evidence base. Most published research originates from high‐income Western countries, which limits the generalisability of findings to diverse global populations, where cultural and healthcare system factor may be influential on CRF experiences and needs [17]. Similarly, the overrepresentation of breast, head and neck and prostate cancers (collectively 83% of studies) omits other prevalent cancers, such as colorectal or lung cancer, which may have unique fatigue profiles. For occupational therapists, this lack of diversity complicates the selection of outcome measures that are broadly applicable across patient populations, indicating a need for inclusion of underrepresented groups and settings in future research.

Although the NCCN recommends a broader range of fatigue assessment tools [1], only five of these were identified in this review. This likely reflects the specific inclusion criteria applied, including a focus on studies conducted in outpatient RT settings and those used within clinical practice contexts. As a result, some recommended measures have not been captured. This represents a limitation of the review and highlights a potential gap between recommended tools and those currently used in practice.

The studies also reflect choice made by researchers, within a research context. For example, the original (40 items) and revised (22 items) versions of the PFS were utilised in three studies [30–32]. However, a brief 12‐item version of the tool has been available for over a decade [47] and covers all four dimensions of CRF—general, physical, cognitive and emotional fatigue [48]. Confusingly, both the 12‐ and 22‐item versions of this scale are called the revised PFS in the literature. Its brevity may make it more suitable to clinical contexts, but it may not collect sufficient information from the research point of view. This gap between what appears in the evidence based and the actuality of clinical practice adds complexity to translating these findings into occupational therapy practice.

Regarding the review methodology adopted, the restriction to English‐language publications also contributes to the previously noted geographical bias. The exclusion of grey literature may also have resulted in relevant tools or emerging evidence not being captured. The inclusion of studies published after January 2010 ensured currency but may have omitted earlier foundational work on CRF outcome measures including tools which remain in clinical use. Although the search was conducted in 2025 and included studies published up to 31 December 2024, no eligible studies published after 2022 met the inclusion criteria. This likely reflects the narrow clinical focus of the review on validated CRF outcome measures applied in outpatient RT settings, rather than a lack of contemporary research relating to CRF more broadly. As a result, newer studies conducted in broader oncology populations or nonclinical contexts may not have been captured.

The exclusion of studies focused solely on psychometric testing or nonclinical settings ensured a focus on practical utility but may have overlooked emerging tools in development or those validated in controlled settings. In addition, the lack of formal quality appraisal, while consistent with scoping review methodology [22], means that the rigour of included studies was not evaluated which may affect the reliability of the findings.

This review also adopts an occupational therapy perspective, which emphasises the importance of activity and participation in assessing CRF. This lens may not align with the primary purpose of many fatigue outcome measures, which are generally designed to assess symptom severity rather than functional impact. The finding that participation is an important gap in existing measures therefore reflects a discipline specific perspective which may not be more broadly relevant across clinical or research contexts. The review also did not formally evaluate how participation is (or should be) operationalised within fatigue measures, which may vary significantly depending on context and patient population.

## 5. Conclusion

Current fatigue outcome measures rarely capture the occupational dimensions of CRF and do not extend beyond what standard occupational therapy activity and participation measures already assess. Occupational therapists should therefore use existing tools judiciously, combining them with occupation focused assessment to inform care. Future research should prioritise codesigning and validating measures that capture how fatigue shapes engagement in daily life, roles and routines across diverse populations. Developing such tools will enable occupational therapists to evaluate interventions and advocate for practice grounded in occupation and participation.

## Funding

Open‐access publishing was facilitated by Deakin University, as part of the Wiley–Deakin University agreement via the Council of Australasian University Librarians.

## Conflicts of Interest

The authors declare no conflicts of interest.

## Supporting information


**Supporting Information** Additional supporting information can be found online in the Supporting Information section. Detailed search strategy used for the MEDLINE database search conducted on 26 March 2025.

## Data Availability

Data sharing is not applicable to this article as no datasets were generated or analysed during the current study.
