# Supplementary figures and images for: Outcome Measures of Fatigue in Adults With Cancer Receiving Radiation Therapy: A Scoping Review
Source: Occup Ther Int. 2026 Jun 1;2026:7068284. doi: 10.1155/oti/7068284 (PMC13239399; doi:10.1155/oti/7068284)

**Supplementary Material 1 – Search Strategy**

MEDLINE

Date: 26th March 2025


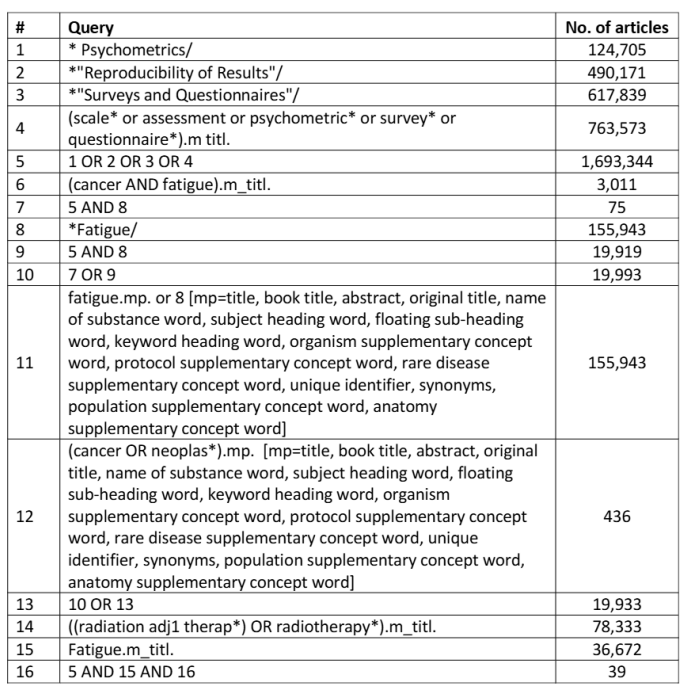

Supplement: Supplementary file 1 — Supporting Information Additional supporting information can be found online in the Supporting Information section. Detailed search strategy used for the MEDLINE database search conducted on 26 March 2025. [file OTI-2026-7068284-s001.docx]
